# Psychological Adverse Effects of Antipsychotic Medication after Remission of First Episode Psychosis: a HAMLETT Ecological Momentary Assessment Study

**DOI:** 10.1192/j.eurpsy.2025.2073

**Published:** 2025-08-26

**Authors:** M. Djordjevic, S. S. Gangadin, L. de Haan, H. E. Jongsma, P. P. Oomen, C. J. Simons, M. Marcelis, M. Nijland, M. J. Begemann, I. E. Sommer, M. Kikkert, W. Veling

**Affiliations:** 1 Department of Psychiatry, University Medical Center Groningen (UMCG), Groningen; 2 Department of Psychiatry, Amsterdam University Medical Center (Amsterdam UMC), Amsterdam; 3 Department of Psychiatry and Neuropsychology, Maastricht University, Maastricht; 4 Department of Research, Arkin Mental Health Care, Amsterdam, Netherlands

## Abstract

**Introduction:**

Current evidence on psychological adverse effects (AEs) of antipsychotic medication after remission of First Episode Psychosis (FEP), and the impact of these AEs on daily life, is limited.

**Objectives:**

To investigate serial cross-sectional associations between antipsychotic medication regimen and psychological AEs after remission of FEP.

**Methods:**

This Ecological Momentary Assessment (EMA) study investigates baseline data of 56 participants from the HAMLETT trial (Handling Antipsychotic Medication: Long-term Evaluation of Targeted Treatment). Momentary mental states indicative of blunted affect intensity and variability, reduced initiative of social contact, avolition and tiredness were assessed 10x/day for eight consecutive days. Based on neurobiological mechanisms likely mediating these psychological AEs, antipsychotic medications were grouped based on their Dopamine-2 (D_2_) and Histamine-1 (H_1_) receptor profile. Multilevel mixed-effects regression models were employed overall and separately for mornings, daytimes and evenings, to investigate serial cross-sectional associations between medication type or dosage and concurrent psychological AEs. All models were adjusted for fixed effects of age, gender, tobacco and cannabis use in the past month and symptom severity during FEP (based on the Comprehensive Assessment of Symptoms and History, CASH).

**Results:**

In total, 85 out of 453 HAMLETT-participants took part in the EMA add-on study. At baseline, 56 (66%) of those participants completed >26 EMA questionnaires and were currently taking antipsychotic medication, yielding a total of 3,005 questionnaires for our analyses. The distribution of antipsychotic medication regimens was relatively equally spread (25% high affinity D_2_ antagonists, 48% low affinity D_2_ antagonists, 27% partial D_2_ agonists). Higher dosage (Beta (B) = -1.11 [95% Confidence Interval (CI): -1.97; -0.24]) and use of high affinity D_2_ antagonists, as compared with partial D_2_ agonists (B = 12.98 [95%CI: 2.43; 23.53]) and low affinity D_2_ antagonists (B = 10.04 [95% CI: 0.59; 19.49]), were associated with decreased positive affect (PA) (see Figure 1). Higher dosage was also associated with small increases in PA variability (B = 0.23 [95% CI: 0.04; 0.42]. The remaining psychological AEs were not associated with dosage or D_2_ profile, neither was H_1_ profile associated with these AEs. Results were relatively consistent across daytimes, though effect sizes were greatest in the evenings.

**Image:**

**Figure:**
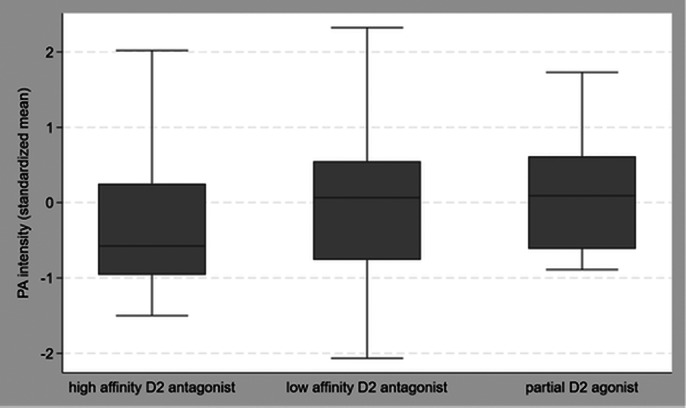

**Conclusions:**

After remission of FEP, higher dosage of antipsychotic medication and use of high affinity D_2_ antagonists, as compared with partial D_2_ agonists and low affinity D_2_ antagonists, can be associated with decreased, though not invariable, positive affect as estimated using EMA.

**Disclosure of Interest:**

None Declared

